# Factors of caregiver support under integrated care for older adults in rural areas: a scoping review

**DOI:** 10.3389/fpubh.2026.1801277

**Published:** 2026-04-02

**Authors:** Chaolumen Bao, Haibo Liu, Thammarat Marohabutr

**Affiliations:** 1 The Affiliated Hospital of Inner Mongolia Medical University, Hohhot, China; 2 Faculty of Social Sciences and Humanities, Mahidol University, Nakhon Pathom, Thailand

**Keywords:** caregiver support, integrated care, older adults, population aging, rural health

## Abstract

**Background:**

Rapid population aging has increased the demand for integrated care for older adults, particularly in rural areas where older adults frequently experience chronic illness, frailty, and limited access to healthcare. Caregivers, both formal and informal, play a central role in supporting the wellbeing of older adults but often face substantial physical, emotional, and financial burdens. Understanding caregiver support is therefore essential to strengthening integrated care for rural older adult populations.

**Objective:**

To identify factors influencing caregiver support within integrated care for older adults in rural areas and to highlight evidence gaps for future research.

**Methods:**

A scoping review was conducted following the Arksey and O’Malley framework. Four databases (PubMed, Scopus, Wiley Online Library, and Taylor & Francis) were searched for English-language studies published between 2014 and 2024. Of 371 records identified, 25 studies met the inclusion criteria and were analyzed by research type, methodology, caregiver type, and caregiver support factors.

**Results:**

Nineteen caregiver support factors were identified and categorized into five domains: social and community, financial, e-health, policy, and spatial. Key challenges included limited education and training opportunities, financial strain, restricted access to telehealth services, inadequate policy support, and transportation barriers. Informal caregivers, primarily family members, predominated in rural settings and experienced greater caregiving burdens due to limited institutional and service support.

**Conclusion:**

Caregiver support is a critical component of effective integrated care for older adults in rural areas. Strengthening social and community networks, financial assistance, e-health infrastructure, and policy frameworks is essential to reducing caregiver burden and improving outcomes for older adults. Future research should prioritize context-specific caregiver support strategies tailored to rural settings.

## 1 Introduction

Population aging has become a major global challenge as the world’s population is aging at an unprecedented pace. Advances in health care, improved survival, and declining fertility rates have driven this profound demographic shift, resulting in people living longer and a growing proportion of older adults worldwide (1). In 2019, more than 1 billion people globally were aged 60 years and older; this number is projected to increase to 1.4 billion by 2030 and further to 2.1 billion by 2050. This rapid growth is expected to accelerate in the coming decades, particularly in developing countries (2).

The transition toward an aging population has significant social and economic implications for many countries (1). As life expectancy increases, the risk of chronic diseases and functional decline rises substantially, placing growing pressure on families and societies to allocate medical, rehabilitative, and long-term care resources (3). Many older adults experience fragmented or inadequate care, particularly those living with multimorbidity and frailty (4). This situation is evident globally. In Australia, more than 83% of individuals aged over 75 years live with two or more chronic conditions (5). In China, nearly 210 million people are aged over 65 years, and more than 78% of them have at least one chronic disease (6). In the United States, approximately half of the population aged over 75 years has three or more chronic conditions (7). Older adults with multimorbidity often face both health and functional challenges, experiencing nearly twice as many care-related problems compared to the general population (8). Frequent visits to multiple healthcare providers, polypharmacy, and care across different healthcare facilities contribute to higher hospitalization rates, increased healthcare utilization and costs, poorer health outcomes, and reduced quality of life (9).

Integrated care has been widely recognized as a key approach to addressing the complex needs of aging populations. Integrated care is defined as an approach that enhances person-centered health systems by ensuring the comprehensive delivery of high-quality services throughout an individual’s life course. It tailors care to the multidimensional needs of individuals and populations and is supported by well-coordinated multidisciplinary teams working across different settings and levels of care (10). For older adults, integrated care is particularly important in addressing multimorbidity, functional decline, and fragmented service delivery (11). Evidence suggests that integrated care can reduce avoidable hospitalizations, as poor care transitions and insufficient follow-up are common contributors to hospital re-admissions among older patients (12). In addition, integrated care emphasizes person-centered care, ensuring that interventions align with older adults’ values, preferences, and social contexts (13).

Caregiver support is a critical component of integrated care for older adults. As aging is often accompanied by declining physical and cognitive function, chronic illness, and geriatric syndromes, many older adults rely heavily on caregivers for daily care, emotional support, and medical management. Caregivers may include family members, professional aides, or community volunteers. However, without adequate support, caregivers themselves are at risk of burnout, financial strain, and emotional distress, which can negatively affect the quality and sustainability of care provided (14, 15). Caregiving can be broadly categorized into formal and informal care. Formal caregivers typically provide paid care and have professional or medical training, whereas informal caregivers are unpaid individuals such as family members, friends, neighbors, or close relatives. While formal caregivers receive varying levels of training, informal caregivers often assume extensive caregiving responsibilities with limited or no formal preparation (16). In addition, access to acute, sub-acute, and geriatric care services remains insufficient in rural and remote areas, increasing reliance on informal caregiving (17).

Compared with metropolitan areas, rural communities generally experience higher poverty rates, a larger proportion of older adults, a greater burden of chronic health conditions, higher proportions of uninsured individuals, reduced access to healthcare services, and higher prevalence of certain substance use behaviors (18). Lack of support is a persistent challenge for caregivers in rural settings. It is essential for caregivers to recognize when they need assistance and to know where to seek support, whether from healthcare professionals, therapists, family members, friends, or peer caregivers (19, 20). The demands of caregiving can result in significant physical and emotional strain, with caregivers often experiencing high levels of unmet needs. As care needs increase over time, caregiver stress and unmet needs tend to intensify. Factors such as limited social support, patient physical disability, lower socioeconomic status, and younger caregiver age have been identified as predictors of caregiver distress. Evidence also indicates that caregivers in rural areas provide longer hours of care compared to their urban counterparts (21). Furthermore, caregivers face numerous barriers to accessing financial support, welfare, and benefits, including unclear eligibility criteria, inconsistent policy implementation, complex administrative procedures, and insufficient support for working caregivers (22).

Although previous reviews have examined caregiving challenges and rural health service delivery, few studies have specifically synthesized the factors influencing caregiver support within integrated care systems for older adults in rural areas. Existing literature often focuses on caregiver burden, informal care dynamics, or rural healthcare access separately, without considering how structural, technological, financial, and policy-related factors interact within integrated care contexts.

Given that rural areas face greater challenges in delivering integrated care for older adults, caregiver support represents a critical yet underexplored area. Therefore, this scoping review focuses on caregiver support within integrated care for older adults in rural settings. The research question guiding this review is: “What factors influence caregiver support within integrated care for older adults in rural areas?” This review aims to map existing evidence, enhance understanding of current caregiver support practices, and identify evidence gaps and inform future research.

## 2 Methods

A review protocol was developed prior to conducting this study to guide the research question, eligibility criteria, search strategy, and data analysis. Although the protocol was not formally registered in a public database, the review followed established methodological guidance for scoping reviews and was reported according to the PRISMA-ScR guidelines. During the study selection process, two reviewers independently screened the titles, abstracts, and full texts of the retrieved records. Any disagreements between the reviewers were resolved through discussion, and if necessary, a third reviewer was consulted to reach consensus. This scoping review was conducted in accordance with the methodological framework proposed by Arksey and O’Malley (23). The review process included identifying the research question, identifying relevant literature sources from open-access and subscription-based databases, developing a comprehensive search strategy, selecting studies based on predefined inclusion and exclusion criteria, charting the extracted data, and collating, summarizing, and synthesizing the findings. Table 1 presents the PRISMA-ScR checklist based on Tricco et al. (24), which aims to improve the transparency, completeness, and clarity of reporting in this scoping review.

**Table 1:** PRISMA-ScR checklist.

Topic	PRISMA-ScR item	Description	Reported in section
Title			
Title	1	Identify the report as a scoping review.	Title
Abstract			
Structured summary	2	Provide a structured summary.	Abstract
Introduction			
Rationale	3	Describe the rationale for the review	1 Introduction
Objectives	4	Provide an explicit statement of the objectives and the questions.	1 Introduction
Methods			
Protocol and registration	5	Indicate whether a review protocol exists.	2 Methods
Eligibility criteria	6	Specify characteristics of the sources of evidence used as eligibility criteria.	2.2 Study selection
Information sources	7	Describe all information sources in the search.	2.1 Search strategies and information sources
Search	8	Present the full electronic search strategy.	2.1 Search strategies and information sources
Selection of sources of evidence	9	State the process for selecting sources of evidence.	2.2 Study selection
Data charting process	10	Describe the methods of charting data.	2.3 Data extraction and synthesis
Data items	11	List all variables for which data were sought.	2.4 Data analysis
Critical appraisal of individual sources of evidence	12	If done, explain the rationale for critical appraisal, the methods used, and how the results were applied.	Not applicable
Synthesis of results	13	Describe the methods of handling the data.	2.3 Data extraction and analysis
Results			
Selection of sources of evidence	14	Give numbers of sources of evidence screened.	3.1 Characteristics of included studies
Characteristics of sources of evidence	15	Present characteristics for which data were charted.	3.1 Characteristics of included studies
Critical appraisal within sources of evidence	16	If done, present data on critical appraisal of included sources of evidence.	Not applicable
Results of individual sources of evidence	17	Present charted data.	3.2 Factors of caregiver support under integrated care for older adults in rural areas
Synthesis of results	18	Summarize charting results.	3.2 Factors of caregiver support under integrated care for older adults in rural areas
Discussion			
Summary of evidence	19	Summarize the main results.	4 Discussion
Limitations	20	Discuss the limitations.	5 Limitations
Conclusion	21	Provide a general interpretation of the results.	6 Conclusion
Funding			
Funding	22	Describe sources of funding	Funding

### 2.1 Search strategies and information sources

The PCC mnemonic was used to guide the development of a clear and meaningful title for this scoping review. PCC refers to Population, Concept, and Context (25) and is commonly applied to define the key components of a scoping review question, as well as to establish clear objectives and eligibility criteria (26). The Population component describes relevant participant characteristics, such as age and other inclusion criteria. The Concept identifies the core phenomenon of interest and determines the scope and depth of the review, while the Context refers to the setting in which the concept is examined and may include geographic location, healthcare setting, or relevant cultural factors (27). As PCC-based keywords, the population was defined as “older adults in rural areas,” the concept focused on “caregiver support,” and the context was “integrated care.” To identify appropriate search terms, the search strategy was iteratively tested and refined through pilot searches. The relevance of retrieved articles was assessed and confirmed in consultation with subject matter experts to ensure comprehensive inclusion of relevant terms. Table 2 presents the PCC mnemonic and the corresponding PCC-based keywords and search terms.

**Table 2:** PCC and search terms.

PCC mnemonic	PCC-based keywords	Search terms
Population	Older adults in rural areas	<older adults>, <elderly>, <seniors>, <aged>, <geriatric>, <rural>, <rural area>, <rural district>, <rural setting>
Concept	Caregiver support	<caregiver support>, <caregiving>, <informal caregivers>, <formal caregivers>, <caregiver burden>, <caregiver resources>
Context	Integrated care	<integrated care>, <coordinated care>, <comprehensive care>, <holistic care>, <continue care>, <multidisciplinary care>, <collaborative care>, <healthcare integration>, <combination care>, <long term care>

In this review, integrated care was operationally defined as the coordinated delivery of healthcare and related support services across different providers, disciplines, or levels of care aimed at addressing the complex and long-term needs of older adults. Studies were included if they examined coordinated healthcare delivery, multidisciplinary collaboration, or the integration of health and social care services that involved caregivers in supporting older adults. This operational definition guided the study selection process to ensure consistent and transparent inclusion decisions.

To identify appropriate search terms, the search strategy was iteratively tested and refined through pilot searches in PubMed and Scopus. During this process, preliminary combinations of keywords were assessed to evaluate their ability to retrieve relevant studies. During the pilot search process, additional terms such as “care partner,” “care worker,” “care aide,” and “paid or unpaid caregiver” were initially considered. However, preliminary searches indicated that the majority of relevant studies were already captured using the terms, such as “caregiver,” “formal caregivers,” and “informal caregivers,” which are widely used in the health and social care literature. Therefore, these broader terms were retained in the final search strategy to ensure both sensitivity and manageability of the search results. This iterative process helped refine the final set of search terms used in the database searches.

Search terms were combined using Boolean operators to construct the following search string: (older adults OR elderly OR seniors OR aged OR geriatric) AND (rural OR rural area OR rural district OR rural setting) AND (caregiver support OR caregiving OR informal caregivers OR formal caregivers OR caregiver burden OR caregiver resources) AND (integrated care OR coordinated care OR comprehensive care OR holistic care OR continuous care OR multidisciplinary care OR collaborative care OR healthcare integration OR combined care OR long term care). A systematic search was conducted across four electronic databases including PubMed, Scopus, Wiley Online Library, and Taylor and Francis to capture both medical and social science literature. PubMed and Wiley Online Library primarily index biomedical research, Taylor and Francis focuses on social science publications, and Scopus provides comprehensive multidisciplinary coverage. The final database searches were conducted on April 20, 2025, and the full search strategies for each database are provided in Table 3.

**Table 3:** Full database search strategies.

Database	Search strategy	Final search date
PubMed	(“older adults” OR elderly OR seniors OR aged OR geriatric) AND (rural OR “rural area” OR “rural district” OR “rural setting”) AND (“caregiver support” OR caregiving OR “informal caregivers” OR “formal caregivers” OR “caregiver burden” OR “caregiver resources”) AND (“integrated care” OR “coordinated care” OR “comprehensive care” OR “holistic care” OR “continuous care” OR “multidisciplinary care” OR “collaborative care” OR “healthcare integration” OR “combined care” OR “long-term care”)	20 November 2024
Scopus	(“older adults” OR elderly OR seniors OR aged OR geriatric) AND TITLE-ABS-KEY (rural OR “rural area” OR “rural district” OR “rural setting”) AND TITLE-ABS-KEY (“caregiver support” OR caregiving OR “informal caregivers” OR “formal caregivers” OR “caregiver burden” OR “caregiver resources”) AND TITLE-ABS-KEY (“integrated care” OR “coordinated care” OR “comprehensive care” OR “holistic care” OR “continuous care” OR “multidisciplinary care” OR “collaborative care” OR “healthcare integration” OR “combined care” OR “long-term care”)	20 April 2025
Wiley Online Library	(“older adults” OR elderly OR seniors OR aged OR geriatric) AND (rural OR “rural area” OR “rural district” OR “rural setting”) AND (“caregiver support” OR caregiving OR “informal caregivers” OR “formal caregivers” OR “caregiver burden” OR “caregiver resources”) AND (“integrated care” OR “coordinated care” OR “comprehensive care” OR “holistic care” OR “continuous care” OR “multidisciplinary care” OR “collaborative care” OR “healthcare integration” OR “combined care” OR “long-term care”)	20 December 2024
Taylor & Francis Online	(“older adults” OR elderly OR seniors OR aged OR geriatric) AND (rural OR “rural area” OR “rural district” OR “rural setting”) AND (“caregiver support” OR caregiving OR “informal caregivers” OR “formal caregivers” OR “caregiver burden” OR “caregiver resources”) AND (“integrated care” OR “coordinated care” OR “comprehensive care” OR “holistic care” OR “continuous care” OR “multidisciplinary care” OR “collaborative care” OR “healthcare integration” OR “combined care” OR “long-term care”)	30 December 2024

### 2.2 Study selection

Articles were selected based on predefined inclusion and exclusion criteria. Eligible studies were peer-reviewed primary, secondary, or methodological research articles published in English between January 2014 and December 2024, with full-text availability. Studies were required to focus on caregiver support for older adults in rural areas and to be related to integrated care.

Articles were excluded if they were not published in English, did not target older adults in rural settings, did not address caregiver support or integrated care, or were published outside the specified time frame. Gray literature and non-research publications, including books, reports, reviews, editorials, theses, dissertations, and conference papers, were also excluded.

Gray literature and non-research publications, including books, reports, editorials, theses, dissertations, and conference papers, were excluded to ensure the inclusion of studies that had undergone peer review and provided sufficient methodological detail for data extraction and comparison. While gray literature can provide additional contextual insights, this review focused on peer-reviewed empirical studies in order to maintain methodological consistency and reliability of the evidence base. Review articles were also excluded because the objective of this scoping review was to identify and map primary evidence on factors influencing caregiver support within integrated care for older adults in rural areas. Including review articles could potentially lead to duplication of evidence from primary studies that were already captured through the database searches.

**Figure 1:**
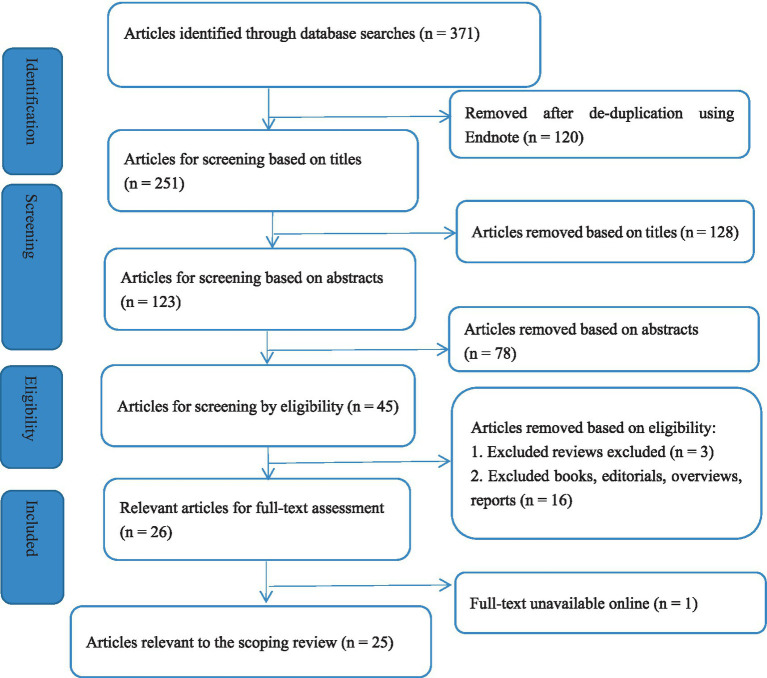
PRISMA-ScR flowchart of process of the literature search and study selection.

A total of 371 records were identified from four databases. After removing 120 duplicates, 251 records were screened by title and abstract, resulting in the exclusion of 206 records. Forty-five full-text articles were assessed for eligibility, of which 20 were excluded due to ineligibility or unavailability of the full text. Ultimately, 25 studies were included in the scoping review, as shown in Figure 1, the PRISMA-ScR flow diagram.

### 2.3 Data extraction and synthesis

Following study selection, relevant data were systematically extracted from the included articles using a predefined charting framework. Extracted information included authors and year of publication, country of origin, research type and design, data collection methods, participant characteristics and sample size, types of caregivers, and factors related to caregiver support within integrated care for older adults in rural areas. The extracted data are summarized in Table 4, which presents the charting of included studies.

**Table 4:** Charting table of the included studies.

Article number	Author and year	Country	Research type	Research design	Data collection	Participants and sample size	Types of caregivers	Factors
1	Brinda et al. (2014) (44)	India	Quantitative	Cross-sectional study	Questionnaires	Dependent older people (*N* = 85) Family caregivers (*N* = 85)	Informal	Cost of caregiving
2	Blusi et al. (2015) (45)	Sweden	Qualitative	Cross-sectional study	Interviews	Family caregivers (*N* = 31)	Informal	Social network
3	Pavarini et al. (2016) (46)	Brazil	Quantitative	Cross-sectional study	Questionnaires	Family caregivers (*N* = 343)	Informal	Education and training support
4	Schaller et al. (2017) (47)	Germany	Qualitative	Cross-sectional study	Questionnaires and interviews	Family caregivers (*N* = 25) Medical professionals (*N* = 2) Social professionals (*N* = 4)	Formal	E-health service
5	Bouldin et al. (2018) (28)	USA	Quantitative	Retrospective study	Secondary data collection	Caregivers (*N* = 7,436)	Informal	Family income
6	Henderson et al. (2018) (42)	Australia	Qualitative	Cross-sectional study	Interviews	Healthcare and social caregivers (*N* = 3 teams)	Formal	Bureaucratization Fragmentation of service delivery Government funding
7	Gibson et al. (2019) (29)	USA	Qualitative	Cross-sectional study	Interviews	Family caregivers (*N* = 11)	Informal	Cost of caregiving Imbalanced medical resources Geographical distance
8	Haya et al. (2019) (51)	Japan	Mixed methods	Cross-sectional study	Questionnaires and interviews	Family caregivers (*N* = 174)	Informal	Family income
9	Lin et al. (2019) (38)	China	Quantitative	Retrospective study	Secondary data collection	1,697 older adults (urban: *N* = 838; rural: *N* = 859)	Formal	Imbalanced medical resources
10	Ko et al. (2020) (30)	USA	Qualitative	Cross-sectional study	Interviews	Family caregivers (*N* = 28)	Informal	Knowledge
11	Schwartz and Jenkin (2020) (31)	USA	Qualitative	Cross-sectional study	Interviews	Old patients (*N* = 8) Caregivers (*N* = 8)	Formal and informal	Cost of treatment Transportation barrier
12	Lewis et al. (2021) (32)	USA	Qualitative	Cross-sectional study	Interviews	Medical caregivers (*N* = 12) Family caregivers (*N* = 22)	Formal and informal	Education and training support Cultural value
13	Longstreth et al. (2022) (33)	USA	Qualitative	Cross-sectional study	Group discussion	Family caregivers (*N* = 175)	Informal	Information
14	Lum et al. (2020) (34)	USA	Qualitative	Descriptive study	Secondary data collection	—	Formal	Telehealth
15	Williamson et al. (2020) (35)	USA	Qualitative	Descriptive study	Secondary data collection	—	Informal	Social workforce 2. Telehealth
16	Aung et al. (2021) (48)	Thailand	Quantitative	Randomized controlled trials method	Questionnaires	Older adults and family caregivers (*N* = 1,734)	Informal	Respite care or day care
17	Disbrow et al. (2021) (36)	USA	Qualitative	Cross-sectional study	Group discussion and interviews	Family caregivers (*N* = 117)	Informal	Education and training support Cost of caregiving
18	George et al. (2022) (43)	Australia	Qualitative	Cross-sectional study	Group discussion	Older adults, professional caregivers, and family caregivers (*N* = 42)	Formal and informal	Knowledge Transportation barrier
19	He et al. (2023) (39)	China	Qualitative	Cross-sectional study	Observation and interviews	Family caregivers (*N* = 24)	Informal	Family income
20	Douglas et al. (2022) (49)	UK	Qualitative	Cross-sectional study	Observation	Community-based multi-disciplinary teams (*N* = 11 teams)	Formal	Multidisciplinary team
21	L’Heureux et al. (2022) (52)	USA	Mixed methods	Cross-sectional study	Interviews	Family caregivers (*N* = 140)	Informal	Social workforce Cost of caregiving
22	Tuttle et al. (2022) (37)	USA	Quantitative	Cross-sectional study	Questionnaires	Family caregivers (*N* = 69)	Informal	Family income
23	Wang and Tang (2023) (40)	China	Mixed methods	Longitudinal study	Secondary data collection	Older adults (2008: *N* = 10,141; 2011: *N* = 6,649; 2014: *N* = 4,886; 2018: *N* = 10,838)	Informal	Cost of caregiving
24	Wu et al. (2023) (41)	China	Qualitative	Cross-sectional study	Interviews	Older adults and professional caregivers (*N* = 33)	Formal	Multidisciplinary team Electronic health record system Government funding Imbalanced medical resources
25	Chambonnière et al. (2024) (50)	France	Qualitative	Cohort study	Questionnaires	Older adults (*N* = 105)	Formal	Education and training support

### 2.4 Data analysis

**Figure 2:**
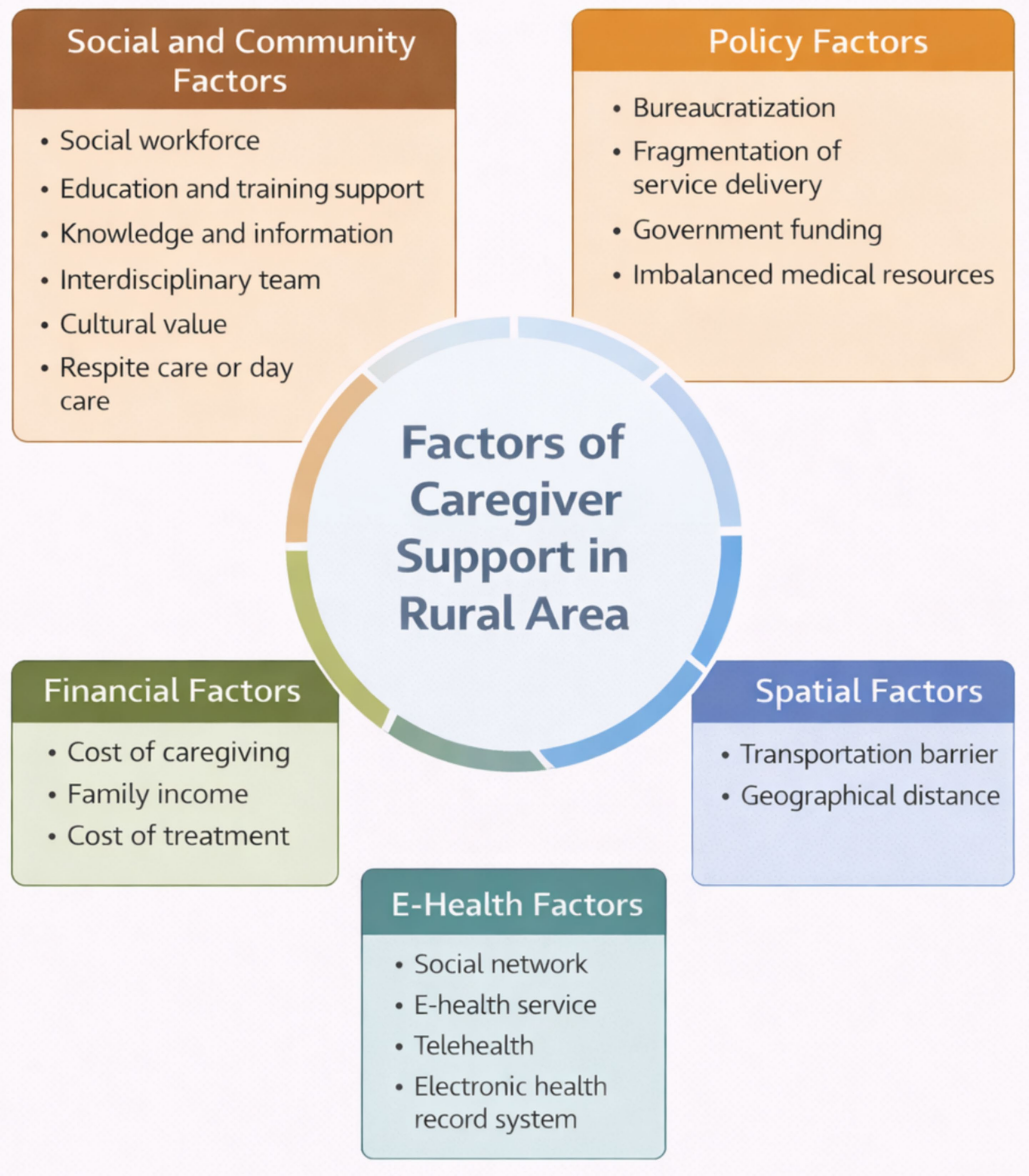
Conceptual framework of factors of caregiver support under integrated care for older adults in rural areas.

Factors reported in the included studies were first extracted and coded as discrete influencing elements. The research team then reviewed these codes and grouped them according to conceptual similarity through an iterative comparison process. During this stage, similar factors were clustered into broader thematic categories. Through discussion and consensus among the researchers, these categories were further organized into five overarching domains: social and community factors, financial factors, e-health factors, policy factors, and spatial factors. This categorization framework was used to structure the synthesis of findings. Figure 2 presents the conceptual framework of factors influencing caregiver support under integrated care for older adults in rural areas.

Consistent with the methodological guidance for scoping reviews from the Joanna Briggs Institute, a formal quality appraisal of the included studies was not conducted (25). The purpose of this scoping review was to map the existing evidence and identify factors influencing caregiver support in rural integrated care contexts rather than to evaluate the methodological quality of the studies or determine the strength of the evidence.

## 3 Results

### 3.1 Characteristics of included studies

A total of 25 studies were included in this scoping review. The characteristics of the included studies were described according to language and country of publication, research type and design, data collection methods, types of caregivers, and factors related to caregiver support within integrated care for older adults in rural areas.

#### 3.1.1 Language and country

All 25 included studies were published in English and originated from 11 countries. Most studies were conducted in the United States (28–37), followed by China (38–41) and Australia (42, 43). One study each was conducted in India (44), Sweden (45), Brazil (46), Germany (47), Thailand (48), the United Kingdom (49), France (50), and Japan (51).

#### 3.1.2 Research types, research design and data collection

In terms of research type, 16 studies employed qualitative methods, six used quantitative approaches, and three applied mixed methods.

Regarding research design, among the qualitative studies, 13 used cross-sectional designs (29–33, 36, 39, 41–43, 45, 47, 49), two employed descriptive designs (34, 35), and one used a cohort design (50). Among the quantitative studies, three applied cross-sectional designs (37, 44, 46), two used retrospective designs (28, 38), and one employed a randomized controlled trial (48). Of the mixed-methods studies, two used cross-sectional designs (51, 52), while one utilized a longitudinal design (40).

In terms of data collection methods, eight studies used interviews (29–32, 38, 41, 42, 45, 51, 52), five used questionnaires (37, 44, 46, 48, 50), and five relied on secondary data sources (28, 34, 35, 38, 40). Two studies employed group discussions (33, 43), two used both questionnaires and interviews (31, 51), one applied observation alone (43), one combined observation and interviews (39), and one used both group discussions and interviews (36).

#### 3.1.3 Types of caregivers

In terms of caregiver type, 15 studies focused on informal caregivers (28–30, 33, 35–37, 39, 40, 44–46, 48, 51, 52). Seven studies examined formal caregivers (34, 38, 41, 42, 47, 49, 50). Three studies addressed both formal and informal caregivers (31, 32, 43).

### 3.2 Factors of caregiver support under integrated care for older adults in rural areas

Based on the reviewed literature, 19 factors related to caregiver support under integrated care for older adults in rural areas were identified across the 25 included studies. These factors were categorized into five domains: social and community factors, financial factors, e-health factors, policy factors, and spatial factors. A summary of these factors is presented in Table 5.

**Table 5:** Factors of caregiver support in the included studies.

Key factors	Specific factors	Article number	
		Formal caregivers	Informal caregivers
Social and community factors	Social workforce	–	15, 21
	Education and training support	25	3, 12, 17
	Knowledge and information	18	11, 13, 18
	Interdisciplinary team	20, 24	–
	Cultural value	12	12
	Respite care or day care	–	16
Financial factors	Cost of caregiving	–	1, 7, 17, 21, 23
	Family income	–	5, 8, 19, 22, 23
	Cost of treatment	10	10
E-health factors	Social network	–	2
	E-health service	–	4
	Telehealth	14	15
	Electronic health record system	24	–
Policy factors	Bureaucratization	6	–
	Fragmentation of service delivery	6	–
	Government funding	6, 24	–
	Imbalanced medical resources	9, 24	7
Spatial factors	Transportation barrier	10, 18	10, 18
	Geographical distance	–	7

#### 3.2.1 Social and community factors

Twelve of the 25 included studies identified social and community factors influencing caregiver support under integrated care for older adults in rural areas. These factors comprised the social care workforce, education and training support, knowledge and information, interdisciplinary teams, cultural values, and respite or day care services.

The social care workforce was highlighted in two studies focusing on informal caregivers. In rural contexts where care is primarily provided by family members, strengthening the professional care workforce and improving access to supportive technologies were reported to enhance caregiver support (29). Social primary care and home care teams were also described as facilitating caregiver self-assessment and linkage to locally available support services (52).

Education and training support was discussed in four studies involving both formal and informal caregivers. Training of professional caregivers improved preventive care activities such as vaccine monitoring and cancer screening among older adults (50), whereas insufficient training among family caregivers contributed to limited medical knowledge, role strain, and challenges in managing disease progression (32, 36). Caregivers in socially vulnerable rural areas were reported to receive lower levels of educational support, which was associated with poorer health and caregiving conditions (46).

Three studies addressed knowledge and information as critical factors affecting both caregiver groups. Limited awareness of available services and care mechanisms negatively influenced caregivers’ experiences with cancer diagnosis and management (43). Among informal caregivers, inadequate knowledge of hospice care impeded medical decision-making (30), while access to referral systems and service linkages was identified as essential for caregivers of older adults with Alzheimer’s disease–related dementias in resource-constrained rural settings (33).

Two studies examined the role of interdisciplinary teams for formal caregivers. Interdisciplinary team-based care was found to promote shared decision-making and patient-centered approaches (41), while community-based teams supported professional caregivers in case management and facilitated integration of health and social care services (49).

Cultural values were identified in one study, emphasizing that shifts in caregivers’ and older adults’ perceptions of Alzheimer’s disease–related dementias are necessary to improve understanding and awareness within inadequately prepared healthcare systems (32).

Finally, respite and day care services were reported in one study as key support mechanisms for informal caregivers, contributing to reduced caregiving burden and strengthening long-term care systems amid increasing non-communicable diseases among rural older adults (48).

#### 3.2.2 Financial factors

Ten of the 25 included studies identified financial factors influencing caregiver support under integrated care for older adults in rural areas. These factors comprised the cost of caregiving, family income, and cost of treatment.

The cost of caregiving was reported in five studies, primarily among informal caregivers. High annual caregiving costs for dependent older adults in rural settings were identified as a major source of caregiver burden, particularly in low- and middle-income countries (44). Substantial financial strain was also reported among family caregivers of older adults with Alzheimer’s disease due to the long-term and specialized nature of care (29, 36). Elevated caregiving costs were observed even among families caring for rural older adults without non-communicable diseases, indicating that financial pressure extends beyond disease-specific care needs (40, 52).

Family income was examined in five studies focusing on informal caregivers. Higher family income facilitated access to additional care resources and improved caregiving capacity (40), whereas low family income was associated with increased caregiver anxiety, employment disruption, and financial stress (51). Rural family caregivers experienced income-related barriers more frequently than urban caregivers (28), with pronounced challenges reported among caregivers of parents with cancer (39). Family income level was consistently identified as a direct contributor to caregiver burden (37).

The cost of treatment was identified in one study, which reported that high treatment costs for geriatric cancer patients limited access to diagnostic procedures, treatments, and follow-up care in rural settings, affecting both formal and informal caregivers (31).

#### 3.2.3 E-health factors

Five of the 25 included studies identified e-health factors influencing caregiver support under integrated care for older adults in rural areas. These factors included social networks, e-health services, telehealth, electronic health record systems, and patient databases.

The role of social networks was reported in one study focusing on informal caregivers. Internet-based social network support was found to reduce loneliness among spousal caregivers in rural areas, thereby improving the effectiveness of geriatric care (45).

One study examined e-health services, describing a dementia care platform that provided tailored support for both formal caregivers and healthcare professionals caring for rural older adults with dementia. Reported benefits included access to personalized information, improved caregiver–professional communication, enhanced remote support, and greater empowerment in health-related decision-making (47).

Telehealth was addressed in two studies. Telehealth-enabled delivery of specialized geriatric consultations was found to be feasible and effective for formal caregivers and older adults in rural settings (34). In addition, telehealth resources improved access to specialized care for informal caregivers of older adults with Alzheimer’s disease–related dementias (35).

The electronic health record system factor was reported in one study involving formal caregivers. Poor integration and limited interoperability of electronic health record systems across healthcare institutions were identified as barriers to effective information sharing and support for rural geriatric care (41).

#### 3.2.4 Policy factors

Four of the 25 included studies addressed policy factors influencing caregiver support under integrated care for older adults in rural areas. These factors included bureaucratization, fragmentation of service delivery, government funding, health insurance, and imbalanced medical resources.

One study reported bureaucratization, fragmentation of service delivery, and government funding as key barriers affecting formal caregivers. Increasing bureaucratization of government-led caregiving services was found to weaken relationships between healthcare and social care providers and to reduce the flexibility required for effective care delivery in rural settings. Fragmentation between federal and state service provision further complicated role clarity for caregivers within integrated care models. In addition, reduced government health budgets resulted in financial shortfalls across caregiving services (42). Another study similarly identified insufficient government funding as a major constraint on formal caregiver support and integrated care implementation (41).

Imbalanced medical resources were identified in three studies involving both formal and informal caregivers. Limited availability of formal caregiving services in rural areas was attributed to increasing demands on already strained healthcare systems (38). Inequitable distribution of healthcare resources was reported to hinder access to professional caregiving support for older adults with chronic conditions (41). This uneven distribution also contributed to unmet needs and restricted access to support services among rural family caregivers (29).

#### 3.2.5 Spatial factors

Three of the 25 included studies addressed spatial factors influencing caregiver support under integrated care for older adults in rural areas. These factors included transportation barriers and geographical distance.

Transportation barriers were reported in two studies involving both formal and informal caregivers. In predominantly rural areas with limited public transportation, older adults with cancer and their caregivers experienced prolonged waits for transportation following treatment, contributing to physical fatigue and care burden (31). Travel to regional or metropolitan centers was also identified as a major challenge to accessing diagnostic services, surgical procedures, certain treatments, and follow-up care for both older adults and their caregivers (43).

Geographical distance was identified in one study focusing on informal caregivers. Long distances between rural communities and essential support services posed significant challenges to family caregivers in accessing appropriate dementia care resources. These distances were reported to limit service availability and disrupt caregivers’ educational and personal development due to the need for extensive travel (29).

## 4 Discussion

The findings of this scoping review indicate that caregiver support for older adults in rural areas can be conceptualized across five interrelated domains: social and community, financial, e-health, policy, and spatial support. Population aging poses a significant challenge that requires coordinated caregiving responses, particularly in rural areas where healthcare and social care resources remain limited. Due to the scarcity of professional long-term care institutions and formal caregiving services in rural settings, combined with the persistent influence of traditional cultural norms, most older adults rely heavily on informal, community- and family-based care. In these contexts, caregiving responsibilities are predominantly assumed by spouses or adult children (39, 51). As a result, strengthening care services and expanding social support networks are essential to alleviating caregiver burden. Actively fostering social networks through caregiver education, empowerment, and encouragement, while supporting caregiver autonomy and independence, is a critical strategy for enhancing caregiver wellbeing (44).

Support from the social workforce is particularly important in improving the physical health and overall wellbeing of family caregivers. Evidence indicates that caregivers in rural areas are more likely to report fair or poor health status and to experience multiple chronic conditions, reflecting broader disparities in health and access to healthcare services compared with urban caregivers (28). Community-based primary healthcare and home care teams are well positioned to identify caregivers’ health needs, assess caregiving responsibilities, and facilitate referrals to appropriate local support services (52). Furthermore, the routine assessment of frailty among caregivers by healthcare professionals has the potential to improve caregivers’ physical health outcomes and prevent further deterioration (50).

Spatial and geographic barriers further exacerbate caregiving challenges in rural areas. Older adults with multiple chronic conditions and their caregivers often face limited transportation options and long distances to healthcare facilities, which restrict access to timely and appropriate care (29, 31, 43). Addressing these challenges requires improvements in transportation infrastructure alongside the expansion of telehealth services, which can facilitate more direct and equitable access to formal healthcare systems for rural populations (34). In addition, the development of interdisciplinary healthcare teams within rural communities, supported by shared electronic patient health records, may enhance care coordination, accessibility, and continuity of services for older adults and their caregivers (41, 49).

Given that families form the backbone of caregiving for older adults in rural areas, education and training represent essential components of caregiver support. Caregivers in rural regions with high social vulnerability often have lower levels of education and receive limited educational and material support for caregiving tasks (46). When care recipients live with multiple non-communicable diseases, family caregivers frequently lack the professional knowledge required to manage complex care needs effectively (36). Establishing rural outreach programs and caregiver support groups, alongside targeted training and education for healthcare providers, is therefore critical. Such initiatives can help address widespread gaps in caregiving knowledge and skills and strengthen the capacity of both caregivers and the rural healthcare workforce (32).

Although the findings of this review were categorized into five domains, these domains are conceptually interconnected and collectively shape caregiver support in rural integrated care settings. Policy factors operate at the macro level and appear to be associated with the regulatory environment, resource allocation, and strategic priorities of integrated care systems. These policy arrangements are linked to financial factors, including funding mechanisms and the affordability of services, which are in turn related to caregivers’ ability to access support resources. Spatial factors reflect the geographical context in which care is delivered. Rural distance, transportation barriers, and the uneven distribution of healthcare facilities is likely associated with limited access to services and greater caregiving burdens. Within this context, e-health factors are connected with potential enabling functions by supporting remote communication, telehealth services, and digital health management, which may help address or alleviate spatial constraints. Social and community factors operate at the community level and appear to be associated with the availability of informal support networks, community resources, and local service coordination. Taken together, these domains are interrelated across policy, structural, technological, and community levels, collectively shaping the sustainability and effectiveness of caregiver support within rural integrated care systems.

Together, these interacting mechanisms highlight that caregiver support in rural integrated care contexts is shaped by a combination of structural, individual, and social processes rather than isolated factors. Therefore, the findings of this review highlight potential associations among factors influencing caregiver support rather than establishing causal relationships.

The findings of this review highlight several policy implications for strengthening caregiver support under integrated care in rural areas. First, policies should focus on strengthening community-based support systems by expanding the social care workforce, improving training programs for caregivers, and promoting interdisciplinary collaboration among health and social care professionals. Second, financial support mechanisms should be enhanced to reduce the economic burden of caregiving, particularly through subsidies, insurance coverage, and targeted financial assistance for low-income households. Third, the development of e-health infrastructure, including telehealth services and electronic health record systems, should be promoted to improve access to healthcare services in geographically dispersed rural areas. Fourth, policymakers should address structural barriers within health systems by improving policy coordination and reducing fragmentation in service delivery. Finally, improving transportation infrastructure and reducing geographical barriers remain critical for enhancing access to integrated care services in rural communities.

## 5 Limitations

This scoping review has several limitations that should be acknowledged. First, although a systematic search was conducted across four widely used academic databases and resulted in the inclusion of 25 relevant studies, literature indexed in other databases may not have been identified. Second, the search strategy, developed through pilot testing, relied on a selected set of caregiving-related keywords, which may have resulted in the omission of relevant studies. For example, studies using alternative terminology (e.g., care partner, care aide) may not have been captured. Third, the review included only studies published in English. Consequently, relevant research published in other languages may have been overlooked, which may have limited the overall comprehensiveness of the findings. Fourth, while this review focused on identifying and synthesizing factors influencing caregiver support in rural integrated care settings, future studies may further interpret these findings using relevant theoretical perspectives, such as health systems theory or the social determinants of health framework, to deepen the understanding of underlying mechanisms. Finally, because a formal quality appraisal was not conducted, the strength and reliability of the individual findings could not be assessed. Despite these limitations, this review employed a rigorous scoping review methodology to systematically map evidence on caregiver support within integrated care for older adults in rural settings.

## 6 Conclusion

This scoping review synthesizes evidence on caregiver support within integrated care for older adults in rural settings across multiple countries, highlighting the central role of caregiver support in sustaining integrated care and promoting older adults’ wellbeing. Despite the five domains of key caregiver support factors identified, important evidence gaps remain. Current studies often acknowledge the need for caregiver education, training, and telehealth support but provide limited guidance on how these interventions should be implemented in rural contexts. While this review identified multiple domains influencing caregiver support in rural integrated care settings, prioritizing these factors based on urgency, modifiability, or systemic importance was beyond the scope of this scoping review. Future research may further evaluate the relative priority of these factors to inform targeted policy development and intervention strategies. Given the interrelated nature of these factors, future research should adopt systematic and context-specific approaches to prioritize and operationalize caregiver support strategies, enabling more effective resource allocation, policy development, and the design of practical, integrated care interventions for rural older adult populations.

## Data Availability

The datasets presented in this study can be found in online repositories. The names of the repository/repositories and accession number(s) can be found in the article/supplementary material.

## References

[1] United Nations Department of Economic and Social Affairs. World Population Ageing 2013. (2013). New York: United Nations.

[2] World Health Organization. Ageing. (2021). Available online at: https://www.who.int/health-topics/ageing#tab=tab_1 (Accessed June 22, 2024).

[3] Zhou Y Li Y Zhu X Ma L. Medical and old-age care integration model and implementation of the integrated care of older people (ICOPE) in China: opportunities and challenges. J Nutr Health Aging. (2021) 25:720–3. doi: 10.1007/s12603-021-1595-5, 34179923 PMC12876801

[4] Shaw S Rosen R Rumbold B. What is Integrated Care? London: Nuffield Trust (2011).

[5] Britt HC Harrison CM Miller GC Knox SA. Prevalence and patterns of multimorbidity in Australia. Med J Aust. (2008) 189:72–7. doi: 10.5694/j.1326-5377.2008.tb01919.x, 18637770

[6] China National Health Commission. The 14th five-year plan healthy aging plan. (2022). Available online at: http://www.nhc.gov.cn/lljks/pqt/202203/c51403dce9f24f5882abe13962732919.shtml (Accessed August 27, 2024).

[7] Wodchis WP Dixon A Anderson GM Goodwin N. Integrating care for older people with complex needs: key insights and lessons from a seven-country cross-case analysis. Int J Integr Care. (2015) 15:e021. doi: 10.5334/ijic.2249, 26528096 PMC4628509

[8] Sarnak DO Ryan J. How high-need patients experience the health care system in nine countries. Issue Brief (Commonw Fund). (2016) 1:1–14. 26809154

[9] Smith SM Soubhi H Fortin M Hudon C O’Dowd T. Managing patients with multimorbidity: systematic review of interventions in primary care and community settings. BMJ. (2012) 345:e5205. doi: 10.1136/bmj.e5205, 22945950 PMC3432635

[10] World Health Organization. Strengthening people-centred health systems in the WHO European region: framework for action on integrated health services delivery. (2016). Available online at: http://www.euro.who.int/__data/assets/pdf_file/0004/315787/66wd15e_FFA_IHSD_160535 (Accessed July 19, 2024).

[11] WHO Clinical Consortium on Healthy Ageing. Report of Consortium Meeting. (2019). New York: United Nations.

[12] Zeynep O Penneau A. A multilevel analysis of the determinants of emergency care visits by the elderly in France. Health Policy. (2018) 122:908–14. doi: 10.1016/j.healthpol.2018.05.003 29807799

[13] Organisation for Economic Co-operation and Development. Integrating care to prevent and manage chronic diseases. (2023). Available online at: https://www.oecd.org/en/publications/integrating-care-to-prevent-and-manage-chronic-diseases_9acc1b1d-en/full-report.html (Accessed September 5, 2024).

[14] Hoffman D Zucker H. A call to preventive action by health care providers and policy makers to support caregivers. Prev Chronic Dis. (2016) 13:E96. doi: 10.5888/pcd13.160233, 27442996 PMC4956477

[15] Prevo L Hajema K Linssen E Kremers S Crutzen R Schneider F. Population characteristics and needs of informal caregivers associated with the risk of perceiving a high burden: a cross-sectional study. Inquiry. (2018) 55:0046958018775570. doi: 10.1177/0046958018775570, 29808748 PMC5977419

[16] Li J Song Y. "Formal and informal care". In: Gu D Dupre ME, editors. Encyclopedia of Gerontology and Population Aging. Cham: Springer Nature (2019). p. 1–8.

[17] Zheng LX Walsh EI Sutarsa IN. Provision of health services for elderly populations in rural and remote areas in Australia: a systematic scoping review. Aust J Rural Health. (2023) 31:805–25. doi: 10.1111/ajr.13016, 37469118

[18] Medline Plus. Rural health concerns. (2023). Available online at: https://medlineplus.gov/ruralhealthconcerns.html (Accessed April 13, 2024).

[19] The Lewin Group. The National Family Caregiver Support Program Resource Guide. Falls Church: The Lewin Group (2022).

[20] Henning-Smith C Lahr M. Rural-urban differences in workplace supports and impacts for employed caregivers. J Rural Health. (2019) 35:49–57. doi: 10.1111/jrh.12309 29949205

[21] Cohen SA Ahmed N Brown MJ Meucci MR Greaney ML. Rural-urban differences in informal caregiving and health-related quality of life. J Rural Health. (2022) 38:442–56. doi: 10.1111/jrh.12581, 33956360

[22] Gardiner C Taylor B Robinson J Gott M. Comparison of financial support for family caregivers of people at the end of life across six countries: a descriptive study. Palliat Med. (2019) 33:1189–211. doi: 10.1177/0269216319861925, 31296108

[23] Arksey H O’Malley L. Scoping studies: towards a methodological framework. Int J Soc Res Methodol. (2005) 8:19–32. doi: 10.1080/1364557032000119616

[24] Tricco AC Lillie E Zarin W O’Brien KK Colquhoun H Levac D . PRISMA extension for scoping reviews (PRISMA-ScR): checklist and explanation. Ann Intern Med. (2018) 169:467–73. doi: 10.7326/M18-0850, 30178033

[25] Peters MDJ Marnie C Tricco AC Pollock D Munn Z Alexander L . Updated methodological guidance for the conduct of scoping reviews. JBI Evid Synth. (2020) 18:2119–26. doi: 10.11124/JBIES-20-00167, 33038124

[26] Pollock D Peters MDJ Khalil H McInerney P Alexander L Tricco AC . Recommendations for the extraction, analysis, and presentation of results in scoping reviews. JBI Evid Synth. (2023) 21:520–32. doi: 10.11124/JBIES-22-00123, 36081365

[27] Aromataris E Lockwood C Porritt K Pilla B Jordan Z. JBI manual for evidence synthesis eds. (2024). Available online at: https://synthesismanual.jbi.global (Accessed March 3, 2024).

[28] Bouldin ED Shaull L Andresen EM Edwards VJ McGuire LC. Financial and health barriers and caregiving-related difficulties among rural and urban caregivers. J Rural Health. (2018) 34:263–74. doi: 10.1111/jrh.12273, 28940539 PMC5866208

[29] Gibson A Holmes SD Fields NL Richardson VE. Providing care for persons with dementia in rural communities: informal caregivers’ perceptions of supports and services. J Gerontol Soc Work. (2019) 62:630–48. doi: 10.1080/01634372.2019.1636332, 31250733

[30] Ko E Fuentes D Singh-Carlson S Nedjat-Haiem F. Challenges and facilitators of hospice decision-making: a retrospective review of family caregivers of home hospice patients in a rural US–Mexico border region—a qualitative study. BMJ Open. (2020) 10:e035634. doi: 10.1136/bmjopen-2019-035634, 32611740 PMC7332198

[31] Schwartz AJ Jenkins CL. Barriers and facilitators to cancer treatment adherence for older rural African Americans: understanding the experience from the view of patients and their caregivers. J Fam Soc Work. (2020) 23:20–34. doi: 10.1080/10522158.2019.1658250

[32] Lewis JP Manson SM Jernigan VB Noonan C. “Making sense of a disease that makes no sense”: understanding Alzheimer’s disease and related disorders among caregivers and providers within Alaska native communities. Gerontologist. (2021) 61:363–73. doi: 10.1093/geront/gnaa102, 32789474 PMC8023374

[33] Longstreth M McKibbin C Steinman B Slosser WA Carrico C. Exploring information and referral needs of individuals with dementias and informal caregivers in rural and remote areas. Clin Gerontol. (2022) 45:808–20. doi: 10.1080/07317115.2019.1710735, 31920164

[34] Lum HD Nearing K Pimentel CB Levy CR Hung WW. Anywhere to anywhere: use of telehealth to increase health care access for older, rural veterans. Public Policy Aging Rep. (2020) 30:12–8. doi: 10.1093/ppar/prz030, 40979974 PMC12447726

[35] Williamson HJ McCarthy MJ Garcia YE Bacon R Dunn DJ Baldwin JA. Addressing the needs of rural caregivers of individuals with Alzheimer’s disease and related dementias during and beyond coronavirus disease 2019 (COVID-19). Public Policy Aging Rep. (2020) 30:178–80. doi: 10.1093/ppar/praa024, 33185627 PMC7499747

[36] Disbrow EA Arnold CL Glassy N Tilly CM Langdon KM Gungor D . Alzheimer disease and related dementia resources: perspectives of African American and Caucasian family caregivers in Northwest Louisiana. J Appl Gerontol. (2021) 40:209–19. doi: 10.1177/0733464820904568 32046583 PMC8637937

[37] Tuttle D Griffiths J Kaunnil A. Predictors of caregiver burden in caregivers of older people with physical disabilities in a rural community. PLoS One. (2022) 17:e0277177. doi: 10.1371/journal.pone.0277177, 36331949 PMC9635719

[38] Lin W. The relationship between formal and informal care among Chinese older adults: based on the 2014 CLHLS dataset. BMC Health Serv Res. (2019) 19:878. doi: 10.1186/s12913-019-4160-8 31118012 PMC6532168

[39] He L Van Heugten K Perez-Y-Perez M Zheng Y. Issues of elder care among migrant workers in contemporary rural China: filial piety redefined from a Foucauldian perspective. J Aging Soc Policy. (2023) 35:554–74. doi: 10.1080/08959420.2021.1926203, 34011242

[40] Wang L Tang Y. Changing trends and the effectiveness of informal care among rural elderly adults in China. SAGE Open. (2023) 13:21582440231202580. doi: 10.1177/21582440231202580

[41] Wu J Xue E Huang S Fu Y Chen D Shao J . Facilitators and barriers of integrated care for older adults with multimorbidity: a descriptive qualitative study. Clin Interv Aging. (2023) 18:1973–83. doi: 10.2147/CIA.S436294 38050622 PMC10693763

[42] Henderson J Dawson S Fuller J O’Kane D Gerace A Oster C . Regional responses to the challenge of delivering integrated care to older people with mental health problems in rural Australia. Aging Ment Health. (2018) 22:1031–7. doi: 10.1080/13607863.2017.1320702, 28463520

[43] George M Smith A Ranmuthugula G Sabesan S. Barriers to accessing, commencing and completing cancer treatment among geriatric patients in rural Australia: a qualitative perspective. Int J Gen Med. (2022) 15:1583–94., 35210830 10.2147/IJGM.S338128 PMC8859537

[44] Brinda EM Rajkumar AP Enemark U Attermann J Jacob KS. Cost and burden of informal caregiving of dependent older people in a rural Indian community. BMC Health Serv Res. (2014) 14:207. doi: 10.1186/1472-6963-14-207, 24886051 PMC4022434

[45] Blusi M Kristiansen L Jong M. Exploring the influence of internet-based caregiver support on experiences of isolation for older spouse caregivers in rural areas: a qualitative interview study. Int J Older People Nursing. (2015) 10:211–20. doi: 10.1111/opn.12074, 25425070

[46] Pavarini SCI Neri AL Brígola AG Ottaviani AC Souza ÉN Rossetti ES . Elderly caregivers living in urban, rural and high social vulnerability contexts. Rev Esc Enferm USP. (2017) 51:e03254. doi: 10.1590/s1980-220x2016040103254, 29211233

[47] Schaller S Marinova-Schmidt V Setzer M Kondylakis H Griebel L Sedlmayr M . Usefulness of a tailored eHealth service for informal caregivers and professionals in the dementia treatment and care setting: the eHealthMonitor dementia portal. JMIR Res Protoc. (2016) 5:e43., 27050401 10.2196/resprot.4354 PMC4822652

[48] Aung TNN Aung MN Moolphate S Koyanagi Y Supakankunti S Yuasa M. Caregiver burden and associated factors for the respite care needs among the family caregivers of community-dwelling senior citizens in Chiang Mai, Northern Thailand. Int J Environ Res Public Health. (2021) 18:5873. doi: 10.3390/ijerph18115873, 34070766 PMC8197883

[49] Douglas N Mays N Al-Haboubi M Manacorda T Thana L Wistow G . Observations of community-based multidisciplinary team meetings in health and social care for older people with long-term conditions in England. BMC Health Serv Res. (2022) 22:758. doi: 10.1186/s12913-022-07971-x, 35676685 PMC9175164

[50] Chambonnière C Blanquet M Delorme C Flory L Metz L Duclos M. Screening for frailty according to rural and suburban health areas in the context of adapted integrated care for older people approach: the FRAGING study. Public Health Nurs. (2024) 42:771–85. doi: 10.1111/phn.13485, 39545458

[51] Haya MAN Ichikawa S Wakabayashi H Takemura Y. Family caregivers’ perspectives on the effect of social support on their care burden and quality of life: a mixed-method study in rural and sub-urban Central Japan. Tohoku J Exp Med. (2019) 247:197–207. doi: 10.1620/tjem.247.197, 30890666

[52] L’Heureux T Parmar J Dobbs B Charles L Tian PGJ Sacrey LA . Rural family caregiving: a closer look at the impacts of health, care work, financial distress, and social loneliness on anxiety. Healthcare (Basel). (2022) 10:1155. doi: 10.3390/healthcare10071155, 35885682 PMC9318565

